# Identification of Context-Specific Fitness Genes Associated With Metabolic Rearrangements for Prognosis and Potential Treatment Targets for Liver Cancer

**DOI:** 10.3389/fgene.2022.863536

**Published:** 2022-05-13

**Authors:** Shizhe Yu, Haoren Wang, Jie Gao, Long Liu, Xiaoyan Sun, Zhihui Wang, Peihao Wen, Xiaoyi Shi, Jihua Shi, Wenzhi Guo, Shuijun Zhang

**Affiliations:** ^1^ Department of Hepatobiliary and Pancreatic Surgery, The First Affiliated Hospital of Zhengzhou University, Zhengzhou, China; ^2^ Henan Engineering Technology Research Center for Organ Transplantation, Zhengzhou, China; ^3^ Zhengzhou Engineering Laboratory for Organ Transplantation Technique and Application, Zhengzhou, China; ^4^ Department of Oncology, The First Affiliated Hospital of Zhengzhou University, Zhengzhou, China

**Keywords:** liver cancer, molecular targeted therapy, CRISPR-Cas9 screens, fitness genes, drug sensitivity, metabolism, trametinib

## Abstract

Liver cancer is the most frequent fatal malignancy. Furthermore, there is a lack of effective therapeutics for this cancer type. To construct a prognostic model for potential beneficiary screens and identify novel treatment targets, we used an adaptive daisy model (ADaM) to identify context-specific fitness genes from the CRISPR-Cas9 screens database, DepMap. Functional analysis and prognostic significance were assessed using data from TCGA and ICGC cohorts, while drug sensitivity analysis was performed using data from the Liver Cancer Model Repository (LIMORE). Finally, a 25-gene prognostic model was established. Patients were then divided into high- and low-risk groups; the high-risk group had a higher stemness index and shorter overall survival time than the low-risk group. The C-index, time-dependent ROC curves, and multivariate Cox regression analysis confirmed the excellent prognostic ability of this model. Functional enrichment analysis revealed the importance of metabolic rearrangements and serine/threonine kinase activity, which could be targeted by trametinib and is the key pathway in regulating liver cancer cell viability. In conclusion, the present study provides a prognostic model for patients with liver cancer and might help in the exploration of novel therapeutic targets to ultimately improve patient outcomes.

## 1 Introduction

Liver cancer, mainly hepatocellular carcinoma (HCC) and intrahepatic cholangiocarcinoma (iCCA), is the leading cause of cancer-related deaths worldwide, with an estimated incidence of over 1 million cases by 2025 (Llovet et al., 2018; Villanueva, 2019; Llovet et al., 2021). Infection with hepatitis B and C viruses, smoking, iron overload, and alcohol-related cirrhosis are well-known risk factors for liver cancer. Recently, metabolic dysregulation in liver cancers has gained more attention, and the associated factors, such as obesity, type 2 diabetes, and non-alcoholic fatty liver disease, have been extensively investigated (Piccinin et al., 2019; Satriano et al., 2019; Faubert et al., 2020).

Owing to the high heterogeneity in liver cancers, systemic therapies, including immune checkpoint inhibitors (ICIs) and tyrosine kinase inhibitors (TKIs), have failed to achieve satisfactory efficacy, especially for patients with advanced stages of the disease (Llovet et al., 2008; Bruix et al., 2017; Kudo et al., 2018; Chen et al., 2019; Bruix et al., 2021; Qin et al., 2021). Accordingly, novel treatment targets must be identified, and patients with liver cancer must be precisely stratified to enable accurate treatment.

Loss-of-function experiments are usually performed during high-throughput screening (HTS), including RNA interference (RNAi) screening (for knockdown) and CRISPR-Cas9 screening (for knockout) for functional genomics and drug discovery (Moffat et al., 2006; Dietzl et al., 2007; Joung et al., 2017). Owing to its high efficiency and specificity, CRISPR-Cas9 screening, which mediates double-strand breaks in the target DNA using guide RNA (gRNA) libraries, is more widely used than RNAi screening (Morgens et al., 2016). There are two main sources of CRISPR screening: the dependency map (DepMap) (Pacini et al., 2021) portal, which is the largest and latest database integrating three large-scale projects, and the BioGRID ORCS (Oughtred et al., 2021), which serves as a warehouse for the published CRISPR screening results. To identify the potential dependency genes that could be used as therapeutic targets, we explored liver cancer datasets deposited in the DepMap database.

Various metabolic alterations occur in liver cancers, such as the upregulation of aerobic glycolysis and nucleotide synthesis, providing energy and biomacromolecules for tumor development and progression (Piccinin et al., 2019; Satriano et al., 2019; Huang et al., 2020). Based on previous studies, metabolic rearrangements, such as the upregulation of nucleotide metabolism and downregulation of lipid metabolism, representing poor prognosis, could also serve as prognostic markers for HCC (Bidkhori et al., 2018; Pascale et al., 2019). Furthermore, the liver, which is the major site for carbohydrate and lipid biosynthesis and amino acid metabolism, is important for maintaining metabolic homeostasis (Gebhardt, 1992; Kietzmann, 2017; Piccinin et al., 2019). As a result, evaluating metabolic rearrangement is essential for understanding liver cancer onset and progression.

Here, we identified fitness genes that could be used as therapeutic targets using data from CRISPR-Cas9 screens and constructed a prognostic model. After evaluating the pathways and biological processes of these genes, we found that they were mainly associated with metabolic rearrangements. Thus, robust targets for liver cancer were identified. Overall, a comprehensive picture of potential fitness genes that are critical for the survival or proliferation of liver cancer cell lines is presented herein. These tumor vulnerabilities could facilitate the development of potential therapeutic targets and ultimately improve patient outcomes.

## 2 Materials and Methods

### Data Sources

The dependence scores of liver cancer cell lines were downloaded from the Dep|Map dataset (Pacini et al., 2021); the scores were obtained following a series of loss-of-function genomic screenings in different cell lines.

The normalized gene-level RNA-seq data and clinical information for 347 patients in TCGA-LIHC cohorts were downloaded from UCSC Xena (https://xenabrowser.net/) using the R package, UCSCXenaTools (Wang and Liu, 2019). Mutation data containing somatic variants were stored in the Mutation Annotation Format (MAF) form and downloaded from the Genomic Data Commons (GDC) (https://portal.gdc.cancer.gov/). To obtain 203 patients in the LIRI-JP validation set, RNA-seq data and related clinicopathological data were downloaded from the ICGC website (https://dcc.icgc.org/projects/LIRI-JP) (Zhang et al., 2019).

### Identification of Viability Vulnerability

A negative score indicates that gene knockout inhibits the survival of a cell line, whereas a positive score indicates that gene knockout promotes survival and proliferation. Cutoff values of 0.5 and −1 were used to define growth-suppressing genes and growth-promoting genes, respectively.

The adaptive daisy model (ADaM) is a semi-supervised algorithm for computing the fuzzy intersection of non-fuzzy sets by adaptively determining the minimum number of sets (Hart et al., 2015; Behan et al., 2019). The ADaM has been used to discriminate core-fitness/context-specific essential genes in large-scale CRISPR-Cas9 screens. Only context-specific essential genes and growth-suppressing genes in liver cancer cell lines were included in the downstream analysis.

Previously known essential genes were obtained from two independent large-scale CRISPR screening studies (Hart et al., 2014; Behan et al., 2019).

### Development and Validation of the Tumor Dependency Signature for Liver Cancer

We selected context-specific essential genes and growth-suppressing genes based on the ADaM analysis to reduce the impact of untargetable, common, and essential life pathways. The cases from TCGA LIHC datasets were used as the training set to establish the LASSO model. Univariate analysis and log-rank tests were used to identify the genes with prognostic ability. For genes with prognostic ability, Cox proportional hazard model (iteration = 1,000) with a lasso penalty was used to identify the best gene model with the R package, “glmnet” (Friedman et al., 2010). The best gene model was used to establish the tumor evolution signature. Thereafter, the concordance (c)-index proposed by Harrell was applied to validate the predictive ability of the signature in all datasets using the “survcomp” R package (Haibe-Kains et al., 2008). A larger C-index indicates a more accurate predictive ability of the model.

### Survival Analysis

Kaplan–Meier (K–M) survival curves were generated to graphically demonstrate the overall survival (OS) of the high- and low-risk groups, which were stratified by the liver cancer dependency signature. The curves were also used to evaluate the prognostic differences between tumor cell clusters. The R package, “survival,” was used for the survival analysis.

### Bioinformatics Analyses

#### 2.1.1 Gene Set Variation Analysis

Gene set variation analysis (GSVA) with a collection of expert-annotated vascular-related gene sets was used to identify pathways and cellular processes that were enriched in different samples. Hallmaker signatures were collected from the Molecular Signatures Database (MSigDB version 5.2, http://bioinf.wehi.edu.au/software/MSigDB/), and a list of metabolic pathways was obtained from the KEGG database (Kanehisa et al., 2021).

#### 2.1.2 Enrichment Analysis

We used significant positive correlation and negative correlation metabolic genes, which were obtained from the Metabolic Atlas (Robinson et al., 2020), with a risk score for the enrichment pathway analysis performed with the functional annotation tool, clusterProfiler (Yu et al., 2012). Gene Ontology (GO) (Ashburner et al., 2000; The Gene Ontology Consortium et al., 2021) and Kyoto Encyclopedia of Genes and Genomes (KEGG) (Kanehisa et al., 2021) terms were identified with a strict cutoff of *p* < 0.01 and a false discovery rate (FDR) of <0.05.

#### 2.1.3 Genome Variation Analysis

The Maftools package was used to illustrate the respective mutation profiling of the two risk group levels *via* a waterfall plot. Differentially mutated genes were identified using the “mafCompare” function, where genes mutated in greater than 5% of the LIHC samples in the TCGA cohort were considered (Mayakonda et al., 2018).

#### 2.1.4 Oncogenic Dedifferentiation Stemness Features Analysis

The mRNAsi index used to match TCGA LIHC cancer datasets was obtained from previous studies (Malta et al., 2018). The risk score of TCGA samples was correlated with the corresponding mRNAsi using the R package, ggstatsplot (Patil, 2021).

#### 2.1.5 Drug Sensitivity Analysis

Information on 90 drug-response matrices and the transcriptome matrix of 81 liver cancer cell models were obtained from the LIMORE database (Qiu et al., 2019). The cell line and drug information can be found on the website (https://www.picb.ac.cn/limore). Spearman’s correlation was used to assess the correlation between drug sensitivity and risk score.

### Statistical Analysis

The “Pheatmap” R package was used to generate heatmaps. Survival analysis was performed using the Kaplan–Meier method, and the prediction performance of the risk model was evaluated using receiver operating characteristic (ROC) and the “time-ROC” R package. Multivariate COX regression analysis was used to investigate the prognostic value of the risk score. Hazard ratios (HRs) and 95% confidence intervals (CIs) were also calculated for each variable. *p* < 0.05 was considered statistically significant at *p* < 0.05. All analyses were conducted using R software version 4.0.2 (https://www.r-project.org/).

**FIGURE 1:**
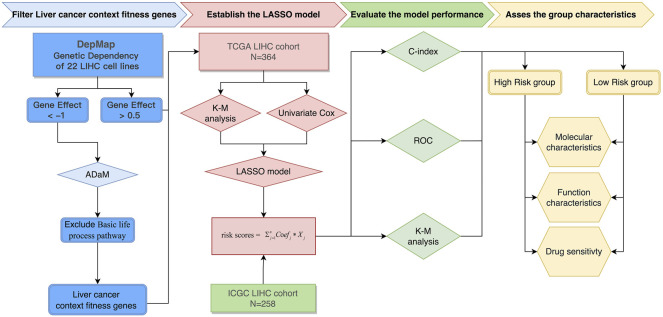
Flowchart of the entire analysis. Flowchart outlining the steps involved in fabricating robust prognostic models from liver cancer context-specific fitness genes.

The entire process for the data analysis is outlined in Figure 1.

## 3 Results

### Identification of Context-Specific Fitness Genes in Liver Cancer Cell Lines

To identify the cancer cell fitness genes (i.e., gene required for cell growth or viability), we performed an integrative analysis of 22 liver cancer cell lines from the DepMap database (Supplementary Table S2). Genes with a dependence score less than −1.0 in at least one liver cancer cell line were defined as fitness genes, while genes with a dependence score more than 0.5 in at least three liver cancer cell lines were defined as suppressor genes. A total of 1.818 fitness genes (Supplementary Figure 1A,B) and 38 suppressor genes were included in the subsequent analysis. Distributions and cumulative distributions of the number of fitness genes were observed in a fixed number of cell lines across 1,000 randomized versions of the depletion scores for the liver cancer cell lines (Supplementary Figure 1C,D).

**FIGURE 2:**
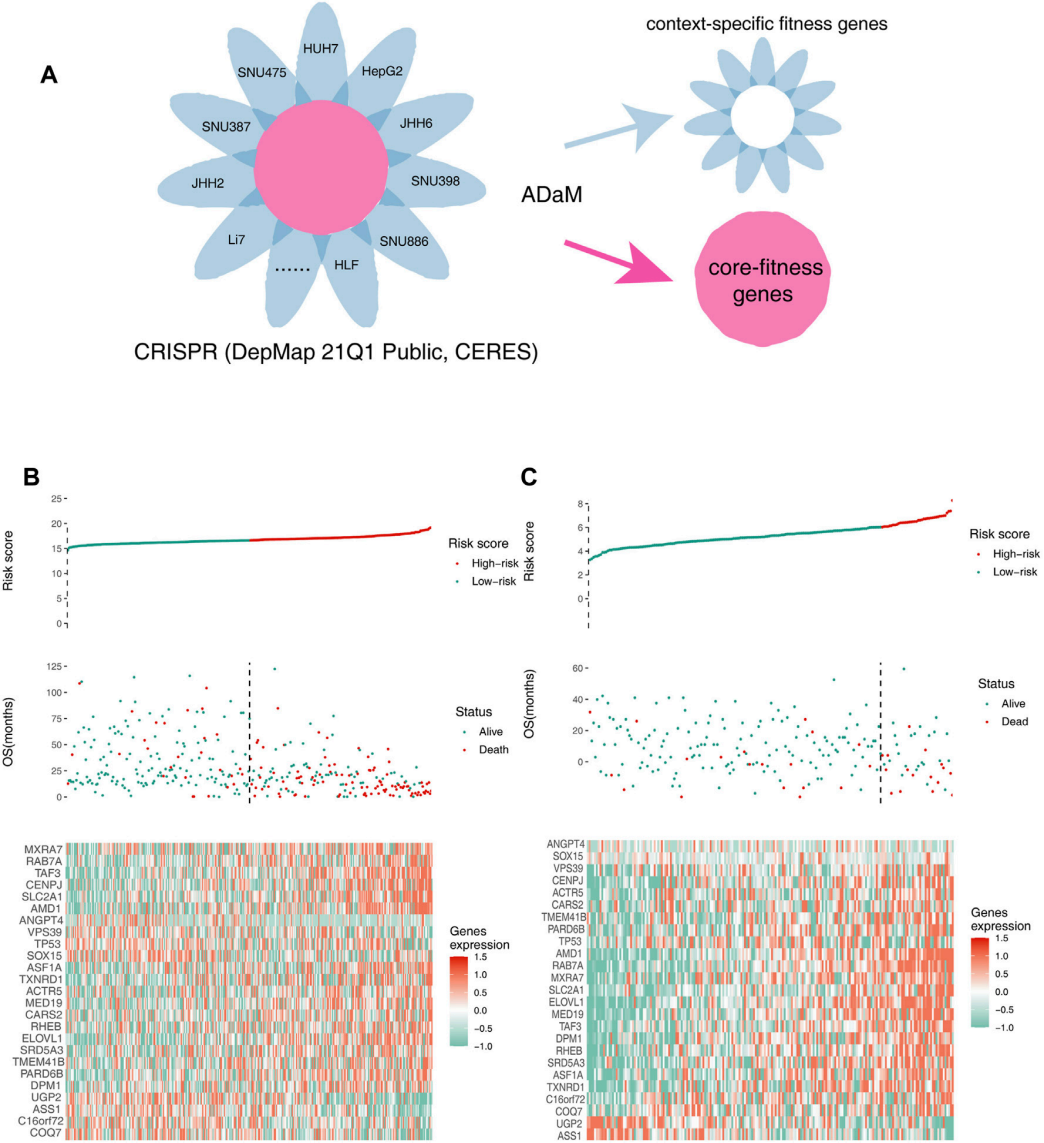
Identifying context-specific fitness genes in liver cancers using the ADaM. **(A)** ADaM distinguished context-specific fitness genes from core fitness genes to select potential targets for liver cancers. **(B,C)** The top graphs show the distribution of risk scores, the center graphs show the survival status of patients in the training and validation cohorts, and the bottom graphs show the expression patterns of the 25 genes. **(B)** TCGA training cohort and **(C)** ICGC validation cohort.

Fitness genes, which are only required for specific molecular or histological contexts, were defined as context-specific fitness genes. In contrast, fitness genes involved in the essential processes of all cells, which had a greater toxicity to normal tissues, were defined as core fitness genes (Figure 2A). To reduce the side effects and select ideal drug targets, context-specific fitness genes must be distinguished from core fitness genes. The ADaM algorithm revealed that the minimum number of dependent cell lines required for a gene to be classified as a core fitness gene is 18. The results were verified using data from the study by Traver Hart et al. and Fiona M. Behan et al., with cover rates of 52 and 63%, respectively, indicating the reliability of our model (Supplementary Figure 1E,F). Genes involved in pathways essential for cell survival were excluded, and 404 context-specific fitness genes were finally obtained for downstream analysis.

To explore the underlying biological functions, functional enrichment analysis was performed. Suppressor genes were found to be enriched in pathways, such as the regulation of cellular response to stress, negative regulation of cell population proliferation, apoptotic signaling pathways, and RHO GTPase effectors (Supplementary Table S1), whereas core fitness genes were enriched in pathways essential for cell survival, such as ribonucleoprotein complex biogenesis, processing of capped intron-containing pre-mRNA, mRNA splicing, RNA transport, DNA replication, and regulation of chromosome organization (Supplementary Table S1). Owing to their lethal side effects, these genes or pathways could not be used as therapeutic targets.

### Construction of the 25-Gene Prognostic Model

To identify survival-related genes and construct a prognostic model, TCGA LIHC data were employed as the training cohort, and ICGC data were employed as the validation cohort. A total of 404 context-specific fitness genes and 38 suppressor genes were used for univariate Cox regression analysis and Kaplan–Meier (K-M) analysis (with *p* < 0.01), respectively, and 202 survival-related genes were initially identified. LASSO Cox regression analysis was used to evaluate the contribution of gene combinations in the training cohort, which revealed a 25-gene signature (Supplementary Figure 1G–I). Based on this signature, samples in the training and validation cohorts were used to calculate the risk scores. Thereafter, patients were divided into high-risk (red) and low-risk groups (green). The OS of the high-risk group was remarkably lower than that of the low-risk group, suggesting a poorer prognosis for the high-risk group (Figure 2B,C). The candidate genes that could be therapeutic targets in this signature is shown in the heatmap (Figure 2B,C).

### Evaluation of the Prognostic Model in the Training Cohort and Validation Cohort

**FIGURE 3:**
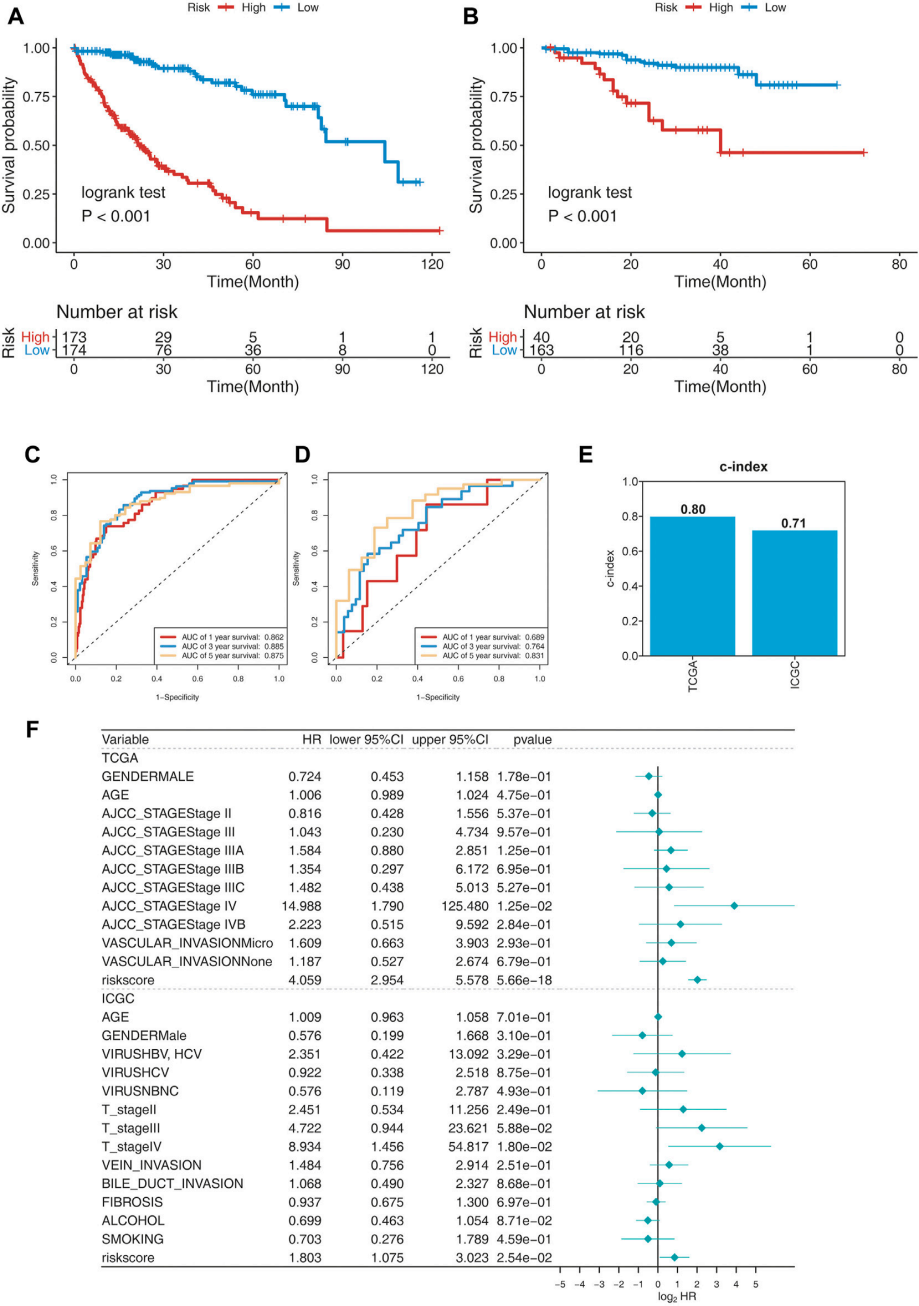
Evaluating the prognosis-predicting efficacy of this 25-gene signature. **(A,B)** Kaplan–Meier plot of TCGA **(A)** and ICGC **(B)** cohorts. **(C,D)** tROC curve of the 25-gene signature in TCGA **(C)** and ICGC **(D)** cohorts. **(E)** C-index of the 25-gene signature was 0.8 in the TCGA cohort and 0.71 in the ICGC cohort. **(F)** Multivariate Cox regression analysis of clinical parameters and risk scores for OS.

To evaluate the prognosis-predicting efficacy of this 25-gene signature, K-M analysis, C-index, and time-dependent ROC (tROC) analysis were performed using TCGA and ICGC cohorts. K-M analysis revealed that patients in the high-risk group had significantly shorter OS than those in the low-risk group in the two cohorts (Figure 3A,B). C-index was performed to validate the credibility of this 25-gene signature, with a value of 0.80 for the TCGA cohort and 0.71 for the ICGC cohort (Figure 3E). The area under the ROC curve (AUC) values for 1-year, 3-year, and 5-year OS were 0.862, 0.885, and 0.875, respectively, for the TCGA cohort, and 0.689, 0.764, and 0.831, respectively, for the ICGC cohort (Figure 3C,D). These findings indicate the high sensitivity and specificity of this 25-gene signature for survival prediction.

Risk scores and clinical parameters, such as sex, age, stage, vascular invasion, fibrosis, alcohol consumption, and smoking history, were included in the multivariate Cox regression analysis. Stage IV and risk scores were identified as independent prognostic factors for OS (Figure 3F). In fact, risk scores were identified to have significant predictive efficacy, with HR of 4.040, 95% CI of 2.946–5.539, and *p* < 0.001 in the TCGA cohort, and HR of 1.918, 95% CI of 1.163–3.164, and *p* < 0.001 in the ICGC cohort.

### Comparison of Genomic Variations in Different Risk Groups

**FIGURE 4:**
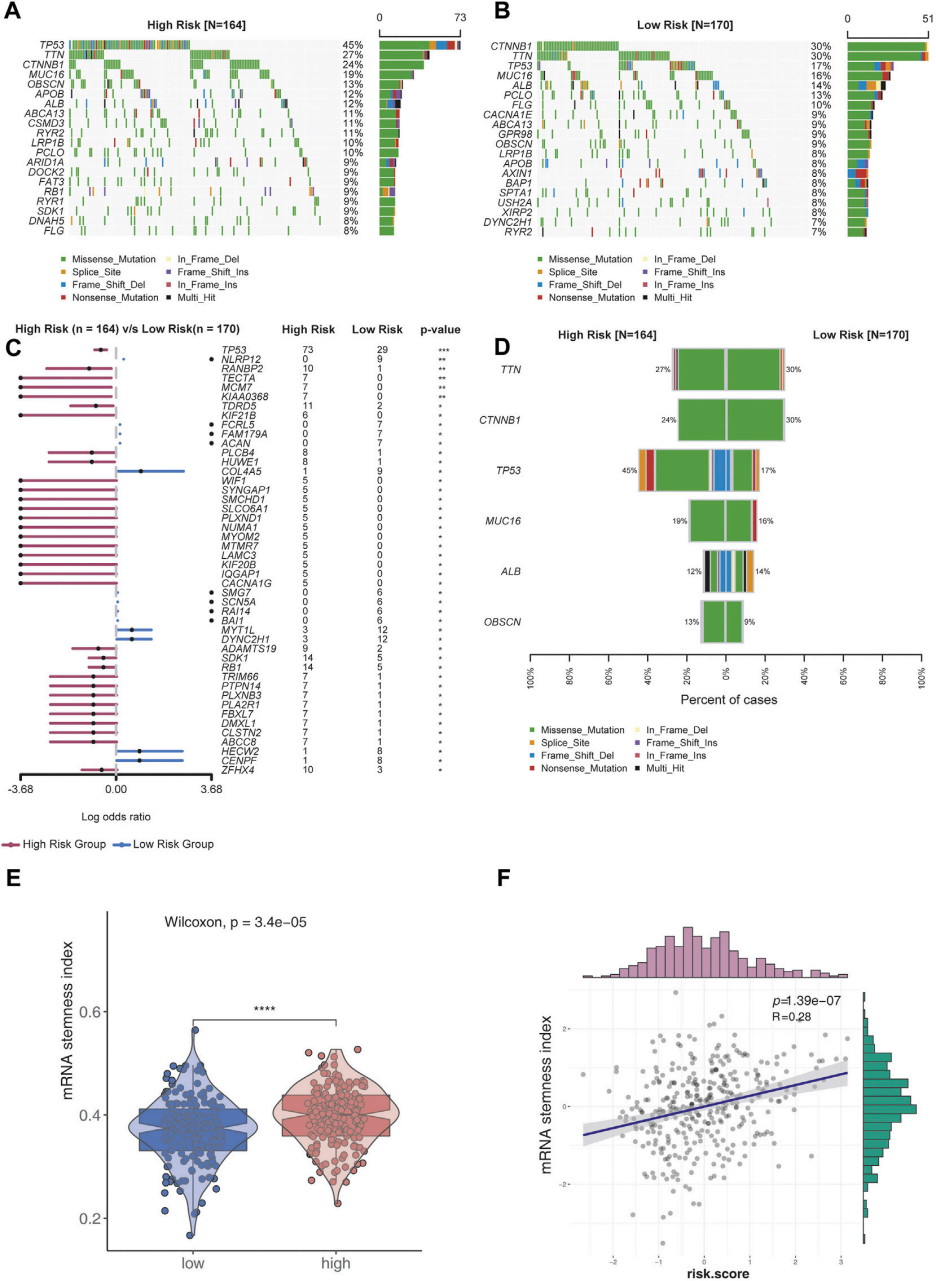
Analysis of genomic variations in the high-risk group and low-risk group. **(A,B)** Oncoplot displaying the somatic landscape of the high-risk **(A)** and low-risk **(B)** groups. Genes were arranged according to their mutation frequencies. The Y-axis represents the gene name, and the abscissa represents the sample name. Different colors represent different mutation types. **(C)** Forest plot showing differentially mutated genes between the high- and low-risk groups. The adjacent table includes the number of samples in the high- and low-risk groups with mutations in the highlighted gene. *p*-value indicates significance threshold: (***) *p* < 0.001, (**) *p* < 0.01, Fisher’s exact test. **(D)** Cobar plots show the most recurrently mutated genes in the high- and low-risk groups. **(E)** mRNA stemness index of the low-risk group was lower than that of the high-risk group. **(F)** mRNA stemness index was positively correlated with risk scores.

The top 20 genes with high genomic mutation frequency in the high-risk and low-risk groups were obtained using Maftools. Furthermore, *TP53* was identified as the most recurrently mutated gene in the high-risk group (45%), while *CTNNB1* was the most recurrently mutated gene in the low-risk group (30%) (Figure 4A,B). To analyze the discrepancy between the high- and low-risk groups, the differentially mutated gene type and frequency were compared using Fisher’s exact test. Most differentially expressed genes were found to be upregulated in the high-risk group, except for *COL4A5* (high-risk vs. low-risk: 1% vs. 9%), *MYT1L* (3% vs. 12%), *DYNC2H1* (3% vs. 12%), *HECW2* (1% vs. 8%), and *CENPF* (1% vs. 8%) (Figure 4C,D).

Stemness is defined as the potential for differentiation from the cell of origin. Stemness is involved in cancer progression, increases the possibility of metastasis and resistance, and results in a poor prognosis. By assessing the degree of oncogenic dedifferentiation in the two risk groups using a one-class logistic regression machine learning algorithm (OCLR), we found that the stemness index was significantly higher in the high-risk group than the low-risk group (Figure 4E). Furthermore, a strong positive correlation was found between the stemness index and risk scores (Figure 4F), indicating higher malignancy in the high-risk group than the low-risk group.

### Exploration of the Biological Processes of This 25-Gene Signature With GSVA

To further explore the underlying biological processes of this 25-gene signature, GSVA was performed with TCGA data. In the Molecular Signature Database (MSigDB) “hallmark” collection of major biological categories, the upregulated genes in the high-risk group were mainly enriched in tumor-promoting and proliferation pathways, such as DNA repair, Myc targets, E2F targets, G2M checkpoint, mitotic spindle, and PI3K/AKT mTOR signaling pathway. Metabolic rearrangement, which plays a pivotal role in oncogenesis, was also found to be significant. Several pathways associated with normal liver function were downregulated, such as oxidative phosphorylation, heme metabolism, bile acid metabolism, peroxisome, adipogenesis, fatty acid metabolism, and xenobiotic metabolism (Supplementary Figure S2A).

Considering the importance of metabolic rearrangement in liver cancers, GSVA was used to explore the KEGG metabolic processes associated with the risk signature. Most functional metabolic pathways in normal livers were found to be downregulated with the risk score (Supplementary Figure S2B, Supplementary Table S3).

### The 25-Gene Signature-Related Metabolic Rearrangement

**FIGURE 5:**
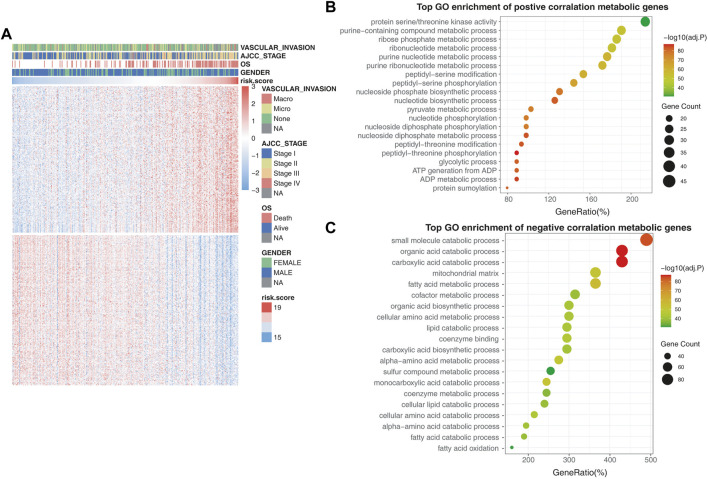
Validating the metabolic rearrangements associated with the prognostic model. **(A)** Differentially metabolic genes in the high-risk group and low-risk group. The heatmap shows that tumor stage and OS were positively correlated with the risk score, while vascular invasion and gender had no relationship with the risk score. **(B)** GO enrichment analysis of the metabolic genes that are positively correlated with risk scores. **(C)** GO enrichment analysis of the metabolic genes that are negatively correlated with risk scores.

To further confirm the role of the 25-gene signature in metabolic rearrangements of liver cancers, metabolism-associated genes were employed from the Metabolic Atlas (Robinson et al., 2020) and 3,625 genes were included for further analysis. A total of 571 genes that were most relevant to the 25-gene signature (Spearman |R| > 0.3, *p* < 0.05) were extracted to generate the heatmap. Of these genes, 282 were significantly positively correlated with the risk scores and 289 were significantly negatively correlated with the risk scores (Figure 5A).

In the GO biological process analysis, the metabolic genes that had a positive correlation with risk scores were mainly enriched in pathways associated with amino acid metabolism and nucleotide metabolism, such as purine-containing compound metabolic process, ribose phosphate metabolic process, ribonucleotide metabolic process, and nucleotide biosynthetic process (Figure 5B). Furthermore, the negatively correlated metabolic genes were mainly enriched in pathways associated with xenobiotic biodegradation metabolism and lipid metabolism, such as small-molecule catabolic processes, organic acid catabolism, and fatty acid metabolic processes (Figure 5C).

### Analysis of Potential Drug Targets With LIMORE

**FIGURE 6:**
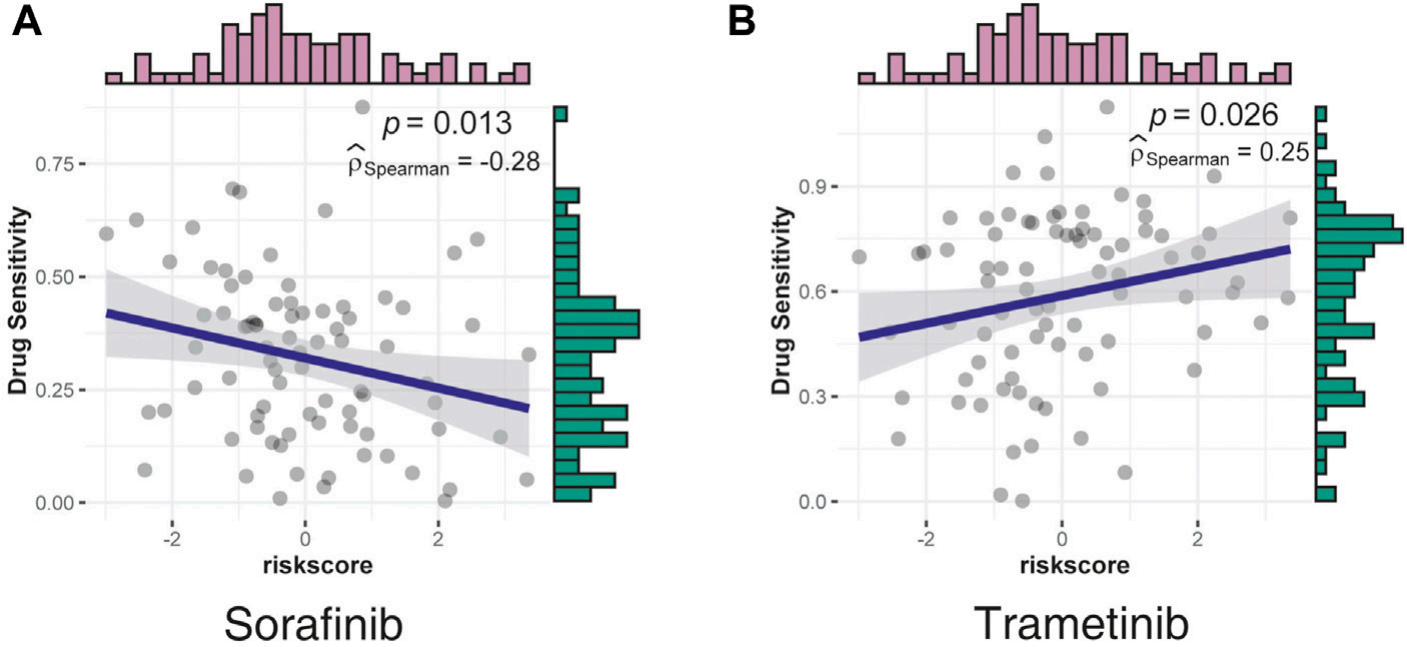
Correlation between drug sensitivity and risk scores was assessed using data from LIMORE. **(A)** The sensitivity of sorafenib had a negative correlation with risk scores. **(B)** The sensitivity of trametinib had a positive correlation with risk scores.

Based on the risk scores, a drug sensitivity analysis was performed using data from LIMORE (Qiu et al., 2019), a pharmacogenomic landscape of human liver cancers. The two drugs with significant correlation—sorafenib, which was negatively correlated with risk scores, and trametinib, which was positively correlated with risk scores—are shown in Figure 6.

Sorafenib, a multi-kinase inhibitor, was the most effective single drug agent for patients with liver cancers for decades; however, this drug only provided survival benefits for 3 months relative to the placebo (Llovet et al., 2008). Consistent with the clinical manifestation, the effect of sorafenib decreased as the risk scores increased, indicating a poor effect in a higher malignancy (Figure 6A).

Consistent with the GO analysis results, which confirmed the serine/threonine kinase activity as the most significant positive correlation pathway, trametinib, a MEK inhibitor mainly used by patients with V600E mutated metastatic melanoma (Robert et al., 2012; Reuters, 2013), was identified as the most positively correlated drug, indicating strong potency in the high-risk group (Figure 6B). However, the underlying mechanisms require further investigation.

## 4 Discussion

Given the global burden of liver cancers and the modest outcomes of current therapeutics, there is a critical need for precise stratified strategies, especially novel comprehensive therapeutic options for patients with liver cancers. In this study, we used the ADaM to identify context-specific fitness genes from DepMap and constructed a 25-gene prognostic model that divided patients into high- and low-risk groups. GSVA and GO analyses revealed significant metabolic rearrangements associated with this signature. Furthermore, a drug sensitivity analysis was performed using data from LIMORE. Genes included in this signature are mainly involved in the life cycle of tumor cells rather than normal cells, suggesting their potential as ideal therapeutic targets with minimal side effects. To date, no studies have systemically explored the role of fitness genes in the treatment of liver cancers. Furthermore, our prognostic model had better predictive power than previous models.

Mutations or dysregulation of transcription factors, essential kinases, and signaling receptors represents a unique class of drug targets that mediate aberrant gene expression, including blocking differentiation and cell death gene expression programs as well as hallmark properties of cancer (Bushweller, 2019). Owing to the cascade amplification effect, minimal perturbations of these upstream molecules can induce significant downstream changes and are at the heart of the overall tumor signaling network (Sever and Brugge, 2015). The differential expression of these core proteins cannot be directly captured using high-throughput analysis. In fact, in conventional transcriptome differential analysis, a minimum threshold of 2-fold expression difference is usually used, and most of the core proteins cannot reach such significant differences. Numerous studies have been conducted on large-sample transcriptome cohorts, such as TCGA, to screen for hub genes (Wang et al., 2021). Owing to the reasons mentioned previously, many upstream core proteins were excluded due to their lower expression and insignificant differential expression. Therefore, a better strategy is needed to identify core proteins that play essential roles in tumors.

The emergence of CRISPR screening technology is an ideal solution to these problems. By performing a large-scale genomic-level lethal gene screen within hundreds of cell lines, the DepMap project could identify core proteins dependent on the growth of tumor cell lines (Pacini et al., 2021). Furthermore, the ADaM was used to select context-specific fitness genes with drug target potential. These genes are proteins that have been validated at a practical level and play a central role in tumor survival. By limiting our targets to these genes, we can effectively avoid the effect of significant signals caused by cascade amplification effects. Combined with the TCGA/ICGC database and DepMap data, targets with both prognostic and therapeutic significance can be effectively screened.

Two problems cannot be avoided if the TCGA database alone is used. First, there is no guarantee that every gene has a significant biological function and may be screened for passenger genes: participants with significant expression changes, but not tumor prognostic differences. Second, the TCGA database may include screening for genes that indirectly affect tumor prognosis, such as PDCD1. Although such genes can also be used as therapeutic targets, this study focused on targeting HCC cells themselves as a killing mechanism.

Of note, only targets with a combination of efficacy and low toxicity are ideal drug targets. Genes that are essential for tumor cell survival but not for the survival of normal tissues should be ideal therapeutic targets with high effectiveness and minimal side effects. Thus, it is particularly important to distinguish context-specific fitness genes from core fitness genes. To explore the genomic effect in improving cancer patient clinical outcomes and accelerate the development of new cancer therapies, CRISPR technologies based on gRNAs have been used to study gene function and identify cellular fitness genes (defined as genes essential for cell growth or viability).

This 25-gene signature based on context-specific fitness genes not only serves as a prognostic model but also provides novel treatment strategies for patients with liver cancer. Most of the genes involved in this signature can be used as therapeutic targets with mild toxicity to normal tissues. Centrosomal P4.1-associated protein (CPAP, also called CENPJ), which is positively correlated with recurrence and vascular invasion in HCC and contributes to sorafenib resistance (Chen et al., 2020), could be a therapeutic target for inhibiting angiogenesis and treating metastatic HCC. Some genes are associated with metabolic processes, such as glucose transporter 1 (*SLC2A1*, also known as *GLUT1*), which encodes a key rate-limiting factor in glucose transport into cancer cells, and thioredoxin reductase 1 (*TXNRD1*), which is the cytosolic subunit and key enzyme of the thioredoxin system. According to previous studies, inhibition of *GLUT1* could impair the growth and migratory potential of HCC cells and reduce glucose uptake and lactate secretion, whereas inhibition of *TXNRD1* hinders the proliferation of HCC cells and induces apoptosis *in vitro* (Amann et al., 2009; Lee et al., 2019). Auranofin (AUR), a pharmacological inhibitor of TXNRD1, can effectively suppress the growth of HCC tumors in animal models and sensitize HCC cells to sorafenib (Lee et al., 2019). Two genes that were negatively correlated with risk scores, UDP-glucose pyrophosphorylase 2 (*UGP2*) and argininosuccinate synthase (*ASS1*), could serve as potential therapeutic targets. In a previous study, the downregulation of *UGP2*, a key enzyme in glycogen biosynthesis, was revealed to be associated with the occurrence and development of various cancer types and poor survival of HCC, while the downregulation of ASS1, a key enzyme in the conversion of nitrogen from ammonia and aspartate to urea, was confirmed to support cancerous proliferation *via* the mammalian target of the rapamycin (mTOR) pathway (Rabinovich et al., 2015; Li et al., 2018; Hu et al., 2020).

Although most therapeutic targets are inhibitors of oncogenes, tumor suppressor genes are more frequently mutated in cancers than oncogenes. With the development of functional genomics and pharmacology, strategies targeting tumor suppressor genes or associated pathways have received increasing attention. Thus, we included the suppressor genes in our analysis. This 25-gene signature could reveal more potential treatment targets for patients with liver cancer.

By evaluating drug sensitivity using data from LIMORE in the present study, we found a positive correlation between the risk scores and the efficacy of trametinib, an MEK inhibitor. A previous study confirmed the potent single-agent antitumor activity of MEK inhibitors in animal models (Huynh et al., 2010; Schmieder et al., 2013). Based on the heterogeneity of liver cancers, the ability of our model to identify the benefits of trametinib is important. These findings indicate that high-throughput drug screens in LIMORE could assess the variable effects of drugs and provide opportunities for pharmacogenomic analysis in liver cancers.

Owing to the high cost of molecularly targeted agents and ICIs, systemic chemotherapy is still an indispensable option, despite its well-known modest efficacy in liver cancers. To reduce side effects, this signature provides a strategy to distinguish the beneficiaries of chemotherapy. GEMOX, based on gemcitabine (GEM) (Mini et al., 2006), an anti-metabolic drug, is a common choice for patients with liver cancer. However, metabolic rearrangement not only promotes the growth and metastasis of tumor cells but also induces GEM resistance. Increasing evidence suggests that gemcitabine resistance is related to the metabolism of glucose, amino acids, and lipids. In this study, the upregulation of GLUT1 promoted glucose uptake and increased glycolysis, resulting in resistance to GEM. In this process, increased glycolytic flux converts glucose intermediates into the pentose phosphate pathway (PPP) and increases pyridine biosynthesis to elevate the intrinsic levels of deoxycytidine triphosphate (dCTP), which competes with the effective levels of GEM (Gu et al., 2021). In addition to the upregulation of GLUT1, increased glycolysis and pyridine biosynthesis metabolism have been confirmed *via* biological analysis. ASS1 is a rate-limiting enzyme involved in arginine synthesis. Owing to a deficiency of ASS1, cancer cells become addicted to external arginine and resistant to GEM (Prudner et al., 2019). Therefore, the high-risk group with significantly higher metabolic rearrangements would be prone to chemoresistance.

Mitogen-activated protein kinases (MAPKs) are protein serine/threonine (Ser/Thr) kinases that convert extracellular stimuli into various cellular responses, including proliferation, migration, differentiation, and metabolism (Yang and Liu, 2017). In the ERK-MAPK signaling pathway, the initiating MAP3Ks are members of the RAF family, which consists of ARARF, BRAF, and CRAF, and are activated by the interaction with active GTP-binding RAS proteins. MAP3K activation leads to the activation of MEK1 and MEK2, which then activate ERK1 and ERK2 through dual phosphorylation of tyrosine and threonine residues (Cargnello and Roux, 2011). After activation, ERK1/2 phosphorylates and triggers a variety of nuclear and non-nuclear proteins that activate proliferative programs and promote the aerobic glycolytic phenotype. Among these transcription factors, c-Myc increases the expression of GLUT1 and several enzymes in the glycolytic pathway, and induces the expression of enzymes involved in nucleotides, fatty acid synthesis, and glutaminolysis (Papa et al., 2019). c-Myc promotes glycolytic intermediates in the PPP, serine, and glycine biosynthesis pathways by inducing the expression of PKM2 (David et al., 2010). These metabolic pathways and serine–threonine kinase activity pathways were found to be upregulated in the high-risk group, indicating a critical role of the ERK–MAPK signaling pathway in HCC.

Sorafenib, initially discovered as a CRAF(BRAF) inhibitor and recently identified as a multikinase inhibitor, has been approved for the treatment of HCC (Llovet et al., 2008). However, the activation of alternative survival pathways leads to drug resistance and limits the effectiveness of sorafenib (Zhou et al., 2019). Of note, the combination of MEK and BRAF inhibitors is better tolerated than their respective monotherapies (Tolcher et al., 2015). The combination of trametinib and dabrafenib, a BRAF inhibitor, was approved for the treatment of *BRAF-V600E/K*-mutant metastatic melanoma in 2014. In this study, the sensitivity to trametinib gradually increased with increasing risk scores, indicating that direct targeting of MEK to block MAPK signaling is an effective option for the treatment of HCC. Previous studies reported no significant improvement in the efficacy of trametinib in combination with sorafenib in patients with unselected HCC, further suggesting the importance of patient selection (Kim et al., 2020). Altogether, our model may provide valuable information for patient selection.

In general, precision medicine could be the only approach to overcome the heterogeneity of liver cancers. This biomarker-driven identification would not only improve therapeutic decision-making but also provide wide therapeutic targets.

## Data Availability

The datasets presented in this study can be found in online repositories. The names of the repositories and accession numbers can be found in section “Materials and Methods.”

## Supplementary Material

Supplementary Figure S1 Establishment of the 25-gene signature. **(A,B)** Number of fitness genes in a fixed number of cell lines. **(C,D)** Distributions and cumulative distributions of fitness genes observed in a fixed number of cell lines across 1,000 randomized versions of the depletion scores for liver cancer cell lines. **(E,F)** Analysis of the data from Hart et al. and Behan et al. was defined as the true-positive rates (blue curve), showing fitness genes in at least 18 cell lines. The possible values of fitness genes in at least 18 cell lines in our study are depicted *via* a red curve. **(G–I)** LASSO regression analysis was used to determine the 25-gene signature for OS prediction: **(G)** Frequency of different gene combination models; **(H)** cross-validation plot showing the confidence intervals for each lambda; **(I)** trajectory of each independent variable; the horizontal axis indicates the log value of the independent variable lambda; and the vertical axis represents the coefficient of the independent variable.

Supplementary Figure S2 GSVA of gene set enrichment in the high-risk group and low-risk group. **(A)** Heatmap of gene set enrichment in the high- and low-risk groups. Differential genes were mainly enriched in the tumor promoting/proliferation pathways and metabolism-associated pathways. **(B)** Heatmap shows metabolic rearrangements in the two risk groups.
