# Supplementary figures and images for: Identification of Context-Specific Fitness Genes Associated With Metabolic Rearrangements for Prognosis and Potential Treatment Targets for Liver Cancer
Source: Front Genet. 2022 May 13;13:863536. doi: 10.3389/fgene.2022.863536 (PMC9136325; doi:10.3389/fgene.2022.863536)

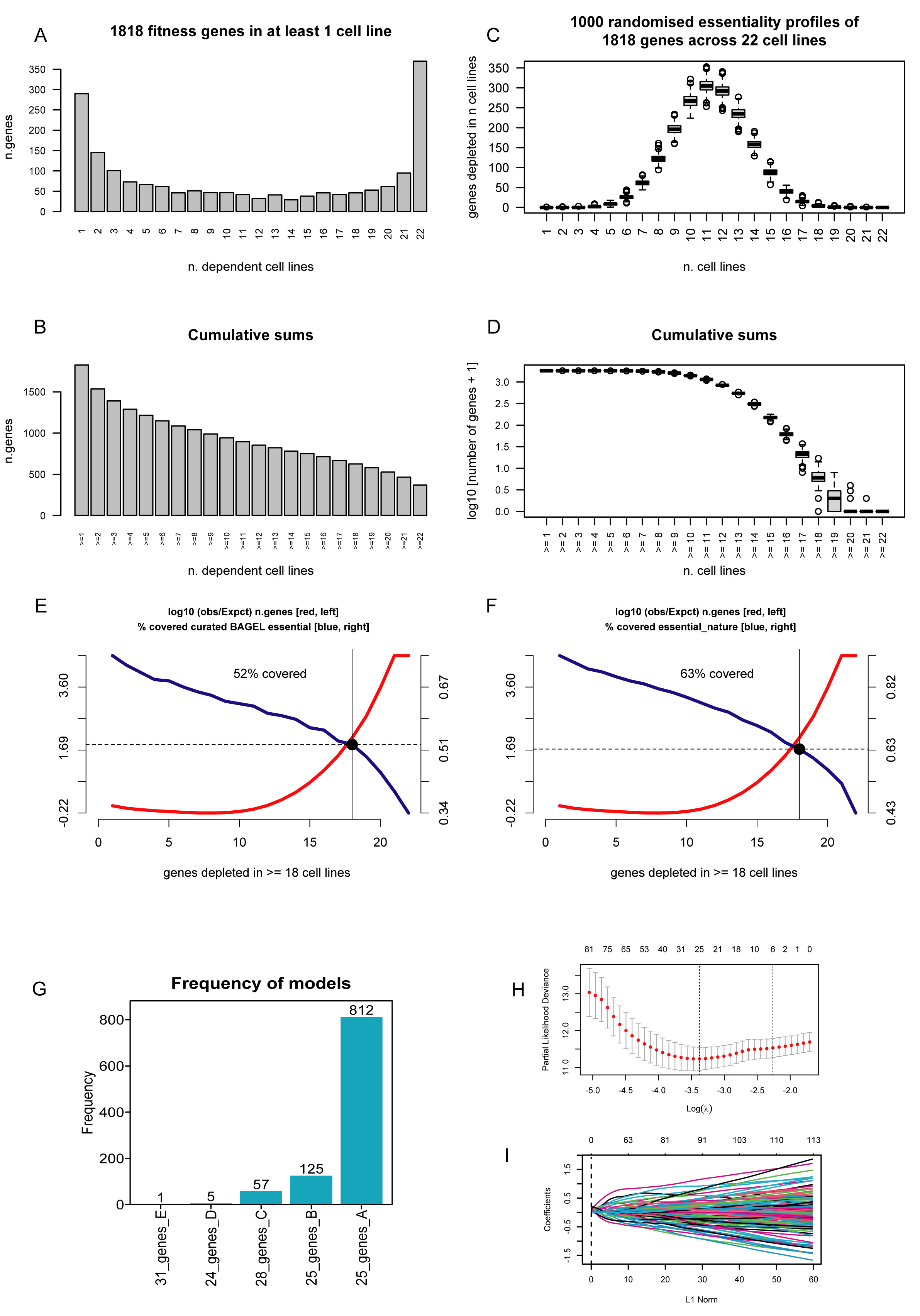

Supplement: Supplementary file 2 [file Image1.JPEG]

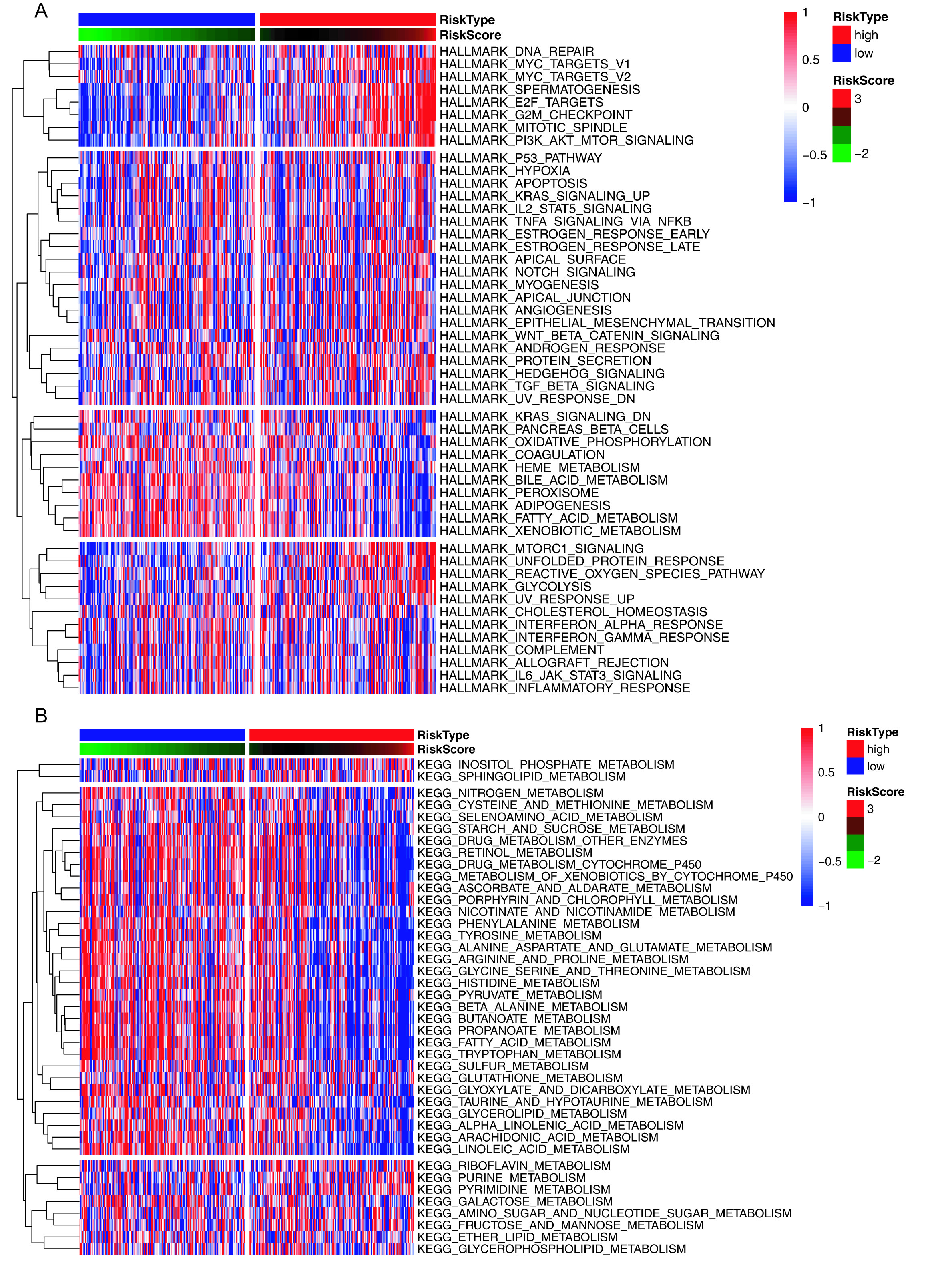

Supplement: Supplementary file 3 [file Image2.JPEG]
